# Slowing of the HIV Epidemic in Ukraine: Evidence from Case Reporting and Key Population Surveys, 2005–2012

**DOI:** 10.1371/journal.pone.0103657

**Published:** 2014-09-24

**Authors:** Charles R. Vitek, Jurja-Ivana Čakalo, Yuri V. Kruglov, Konstantin V. Dumchev, Tetyana O. Salyuk, Ivana Božičević, Andrew L. Baughman, Hilary H. Spindler, Violetta A. Martsynovska, Yuri V. Kobyshcha, Abu S. Abdul-Quader, George W. Rutherford

**Affiliations:** 1 Division of Global HIV/AIDS, Center for Global Health, Centers for Disease Control and Prevention, Kyiv, Ukraine; 2 Division of Global HIV/AIDS, Center for Global Health, Centers for Disease Control and Prevention, Atlanta, Georgia, United States of America; 3 World Health Organization Collaborating Centre for HIV Surveillance, University of Zagreb, Zagreb, Croatia; 4 Ukrainian Center for Socially Dangerous Disease Control of the Ministry of Health of Ukraine, Kyiv, Ukraine; 5 International HIV/AIDS Alliance in Ukraine, Kyiv, Ukraine; 6 Department of Epidemiology and Biostatistics, University of California San Francisco, San Francisco, California, United States of America; 7 World Health Organization, Kyiv, Ukraine; Vanderbilt University, United States of America

## Abstract

**Background:**

Ukraine developed Europe's most severe HIV epidemic due to widespread transmission among persons who inject drugs (PWID). Since 2004, prevention has focused on key populations; antiretroviral therapy (ART) coverage has increased. Recent data show increases in reported HIV cases through 2011, especially attributed to sexual transmission, but also signs of potential epidemic slowing. We conducted a data triangulation exercise to better analyze available data and inform program implementation.

**Methods and Findings:**

We reviewed data for 2005 to 2012 from multiple sources, primarily national HIV case reporting and integrated biobehavioral surveillance (IBBS) studies among key populations. Annually reported HIV cases increased at a progressively slower rate through 2011 with recent increases only among older, more immunosuppressed individuals; cases decreased 2.7% in 2012. Among women <25 years of age, cases attributed to heterosexual transmission and HIV prevalence in antenatal screening declined after 2008. Reported cases among young PWID declined by three-fourths. In 2011, integrated biobehavioral surveillance demonstrated decreased HIV prevalence among young members of key populations compared with 2009. HIV infection among female sex workers (FSW) remains strongly associated with a personal history of injecting drug use (IDU).

**Conclusions:**

This analysis suggests that Ukraine's HIV epidemic has slowed, with decreasing reported cases and older cases predominating among those diagnosed. Recent decreases in cases and in prevalence support decreased incidence among young PWID and women. Trends among heterosexual men and men who have sex with men (MSM) are less clear; further study and enhanced MSM prevention are needed. FSW appear to have stable prevalence with risk strongly associated with IDU. Current trends suggest the Ukrainian epidemic can be contained with enhanced prevention among key populations and increased treatment access.

## Introduction

With the explosive growth of HIV transmission among persons who inject drugs (PWID) in Ukraine beginning in 1995, a wave of HIV infection spread across the former Soviet Union [1]. With continued increases in cases among PWID and, to a lesser degree, other key populations (KP), Ukraine developed the most severe epidemic in Europe and Central Asia with an estimated 440,000 persons living with HIV (PLHIV) in 2007 [2]. Prevention and care responses began with spontaneous local efforts supported by limited local and state budgets and uncoordinated external grants. Beginning in 2004, coordinated external support from the Global Fund, US government, and others allowed a major expansion of prevention programs focused on KP and antiretroviral therapy (ART). In 2011, the number of PWID reached with prevention activities was 160,000 as compared to 54,000 in 2006, while the number of PLHIV on ART increased from 255 in 2004 to>6,000 in 2008 [3]. Since 2008, the government of Ukraine has assumed principal responsibility for ART, which, as of January 2014, reached>55,000 [4] of the estimated 100,000 PLWHA in need of ART [5].

In recent years, available reported data have produced conflicting interpretations of the evolution of the Ukrainian epidemic. While HIV cases among PWID dominated the first decade, heterosexual transmission has been the predominant attributed route of transmission among newly registered HIV cases since 2008 [6]. Annual numbers of newly reported cases progressively increased from 2000 through 2011, although the rate of increase slowed after 2006. The annual number of HIV-positive pregnant women continued to increase through 2011 although HIV prevalence in screening of pregnant women not previously registered as HIV-infected declined after 2009 [6]. To improve the understanding of these changes and better guide the response, we carried out a data triangulation analysis of available HIV surveillance and programmatic data sources for the period 2005–2012. The focus for the analysis reported here was to clarify current patterns of HIV transmission in Ukraine.

## Methods

### Ethics statement

All surveys whose data were included in the analysis had received national institutional review board approval from the Committee on Medical Ethics of the Gromachevsky Institute of Epidemiology and Infectious Diseases and from the Committee of Professional Ethics of the Ukrainian Sociological Society. Case reporting systems are established by Ukrainian law. The analysis was reviewed at the University of California, San Francisco Committee on Human Research and received an exemption due to the lack of access to individual level data.

### Data sources

Triangulation is an analytical approach that compares and interprets multiple data sources to improve the understanding of a public health problem and assess the impact of population-level interventions [7]. Diverse data sources help reduce the random and systematic errors often present in single surveillance and programmatic data sets.

We conducted a secondary analysis of data sources that included HIV case reports, HIV prevalence proxy indicators from antenatal clinic (ANC) and military recruit screening, and HIV prevalence data from integrated biological and behavioral surveys (IBBS) among KP including PWID, female sex workers (FSW) and men who have sex with men (MSM). We also reviewed general population demographic and birth data from the Ukrainian State Statistics Service. No individual level data were reviewed in our analysis; we obtained and analyzed the summary data for these indicators from two sources: 1) for HIV case reports and HIV screening of pregnant women and military recruits, from the Ukrainian Center for Socially Dangerous Disease Control, which houses the national HIV case reporting surveillance system data; and 2) for IBBS surveys, from the International HIV/AIDS Alliance in Ukraine, which coordinates the Global Fund supported national IBBS surveys. Access to this summary data for other researchers is potentially available subject to approval of requests by the respective institutions.

### HIV and AIDS case reports

In Ukraine, population HIV antibody screening, including high coverage screening of pregnant women, has been carried out since 1987. After a positive screening result, individuals are referred to a network of government AIDS centers. These centers are the only providers of HIV care and treatment, which is provided without charge to individuals whose HIV infections are confirmed on supplemental testing; registration is mandatory to receive care. Individual-based data are reported mandatorily for all patients registered in the network; we analyzed summary data reports and restricted our analysis to cases among individuals≥15 years of age. When HIV-infected individuals are registered at AIDS centers, the severity of HIV illness is classified according to a clinical scale based on WHO guidelines [8]. The categories can be grouped into WHO clinical stage I (asymptomatic and persistent generalized lymphadenopathy), clinical stages 2 and 3 symptomatic (mild and advanced), and clinical stage 4 (AIDS-defining conditions). In 2010, newly registered cases with pulmonary TB were no longer classified as stage 3 but were assigned to stage 4. For our analysis, we combined stages 2–4 (symptomatic and AIDS cases) as ‘advanced immunodeficiency’ and compared with stage 1 (‘asymptomatic’). We calculated median ages assuming that cases were evenly distributed in the five- or 10-year age-groups in which they were reported.

Ukraine has 27 administrative subdivisions, including 24 oblasts, Kyiv City, Sebastopol City, and the Autonomous Republic of Crimea; we refer to these generically as regions. We constructed terciles of regions by dividing regions into three groups based on the average annual number of the newly reported HIV cases per 100,000 total population over the period from 2005 to 2012. We refer to these as ‘new case report’ terciles, with the acknowledgment that newly reported cases are not equivalent to newly transmitted cases.

### ANC screening and reported data

All pregnant women not already registered as HIV-positive are provided routine opt-out HIV testing in ANC; screening includes rapid testing for women who present at delivery without antenatal care. HIV status has been identified for>95% of delivering mothers since 2003 and>99% since 2007 (excluding 97.2% in 2010 [6]). Pregnant women who are found to be HIV-infected in ANC screening are registered with AIDS centers where free care and treatment, including antiretrovirals for prevention of mother-to-child transmission (PMTCT), are available. Two types of data were analyzed on HIV prevalence among pregnant women. First, summary data are reported on HIV screening in ANC, which excludes women registered as HIV-infected prior to their pregnancy. Testing twice in pregnancy is recommended. Prior to 2007, only the summary result of all screening HIV tests done in pregnancy was reported; since 2007, data have been reported separately on HIV prevalence at the initial test in pregnancy. Second, summary data are reported on the number of HIV-exposed infants; the mothers of these infants will include both women registered as HIV-infected prior to pregnancy and those identified in ANC. We used the proportion of HIV-exposed births among all births as a proxy for HIV prevalence among delivering mothers.

### IBBS data

Since 2004, IBBS have been conducted by the International HIV/AIDS Alliance in Ukraine, with progressively increasing coverage of KP. All regions were covered for PWID and FSW in the rounds conducted in 2008–2009 (hereafter 2009 round) and 2011, and near-national coverage was first achieved among MSM in 2011. In the 2011 IBBS studies, investigators also estimated hepatitis C (HCV) prevalence, using a combination rapid test for HIV/HCV.

### Statistical analysis

To evaluate trends in reported HIV cases with asymptomatic HIV infection at presentation, among women 15–24 years of age, and among men entering military service, we performed Poisson regression analysis [9] using SAS software [10]. To evaluate trends in HIV prevalence among pregnant women for 2007–2009 and 2010–2012, we used a linear spline Poisson regression model to estimate separate regression lines and perform tests for trends [11]. To compare the proportion of cases with advanced immunodeficiency at presentation between selected years we used Pearson's chi-squared test and to compare HIV seroprevalence in the 2011 FSW survey between FSW subgroups we used Mantel-Haenszel odds ratios. Other statistics reported for survey data are taken from the published reports.

## Results

**Figure 1:**
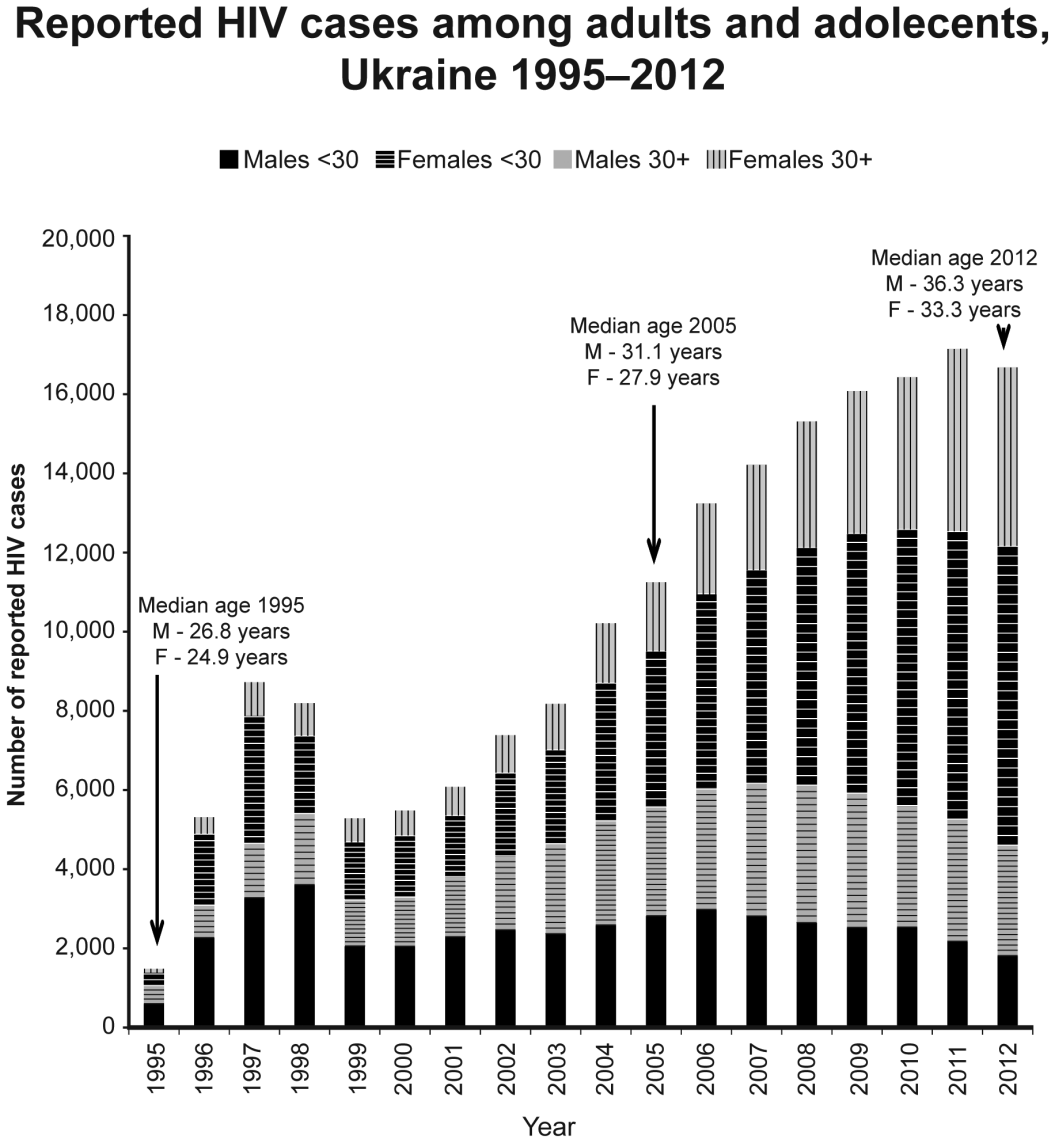
Reported HIV cases among adults and adolescents, Ukraine 1995–2012.

**Figure 2:**
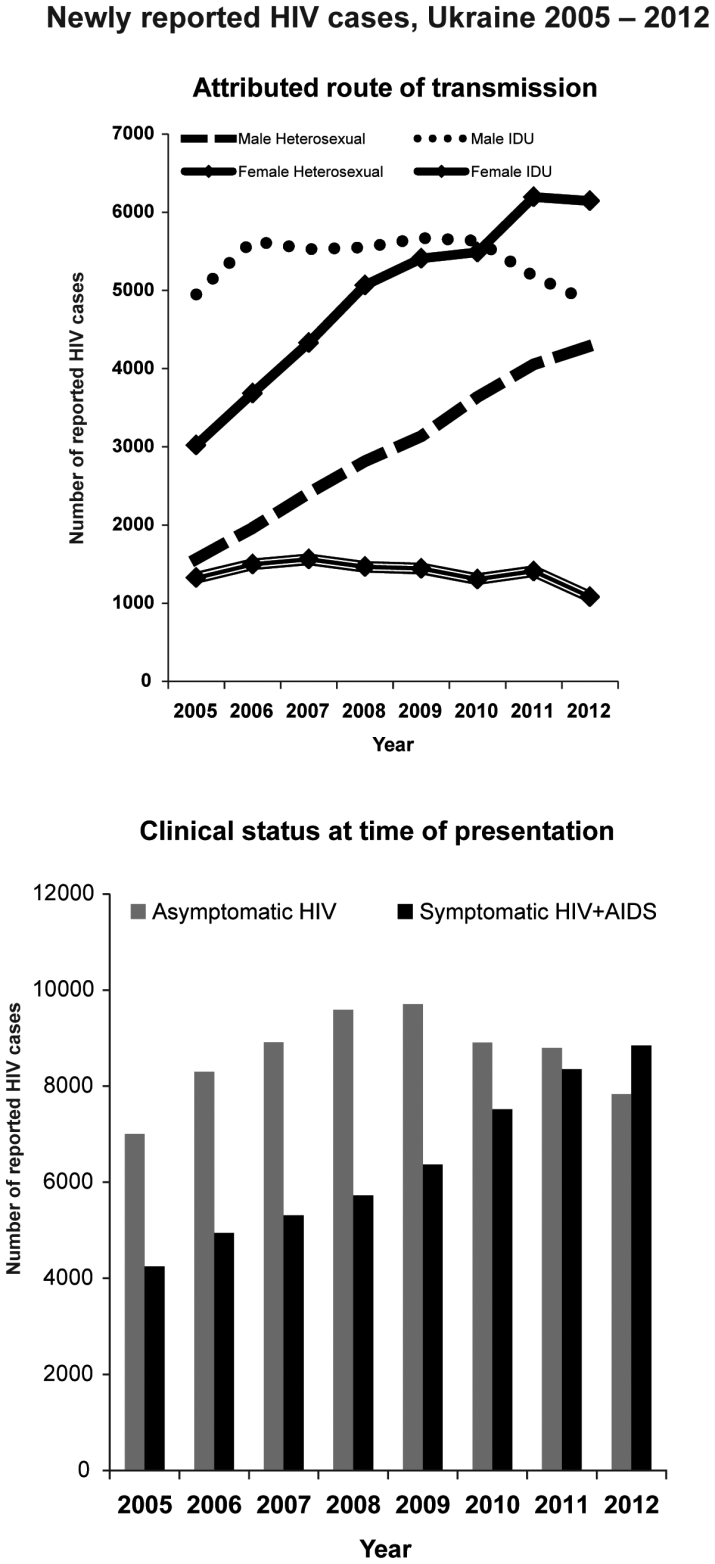
Newly reported HIV cases, Ukraine 2005–2012. a. Attributed route of transmission. b. Clinical status at time of presentation.

From the beginning of case reporting in 1987 until the end of 2012, 186,821 cases of HIV in adults and adolescents≥15 years of age had been reported; from 2005 through 2012, 120,351 cases were reported (**Figure 1**) of which 57.3% were male. The annual rate of increase in reported cases slowed from 10.3% annually in 2005 to 4.4% in 2011; cases then fell by 2.7% in 2012. Reported cases had a median age of 25.9 years in 1995; median age progressively increased from 2005 to 2012 among both men (32.1 to 36.3 years) and women (27.9 to 33.3 years). In recent years, the absolute number of cases reported among individuals <30 years of age has declined both among males (2,993 cases in 2006 to 1,826 in 2012, −39%) and females (3,489 cases in 2008 to 2,781 in 2012, −20%). Since 2007, cases attributed to heterosexual transmission have progressively exceeded those attributed to IDU (**Figure 2a**). The proportion of newly reported adult cases with advanced immunodeficiency at presentation increased sharply from 37.7% in 2005 to 53.0% in 2012 (P<0.01); the absolute number of reported cases with asymptomatic HIV infection at presentation declined by 19.2% from 2009 to 2012 (P<0.01) (**Figure 2b**).

**Figure 3:**
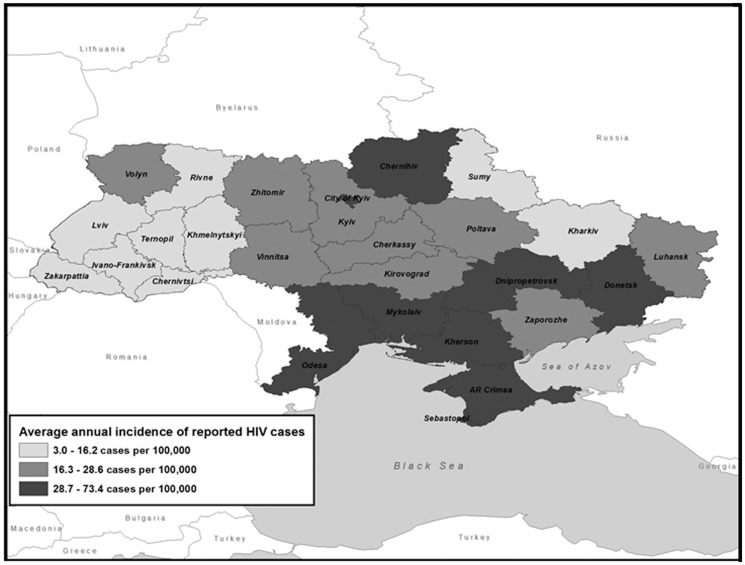
Average annual number of reported HIV cases per 100,000 population, by region, Ukraine 2005–2012.

Reported cases remained highly concentrated in eastern and southeastern regions in 2005–2012 as in previous years (**Figure 3**). The nine regions in the top new case report tercile for all cases contributed 70.1% of HIV cases reported over this period but contained only 40.8% of the population; seven of these regions were in the south and southeast and contributed 61.8% of cases but only 32.3% of the population.

### Trends in reported cases and prevalence among PWID

**Figure 4:**
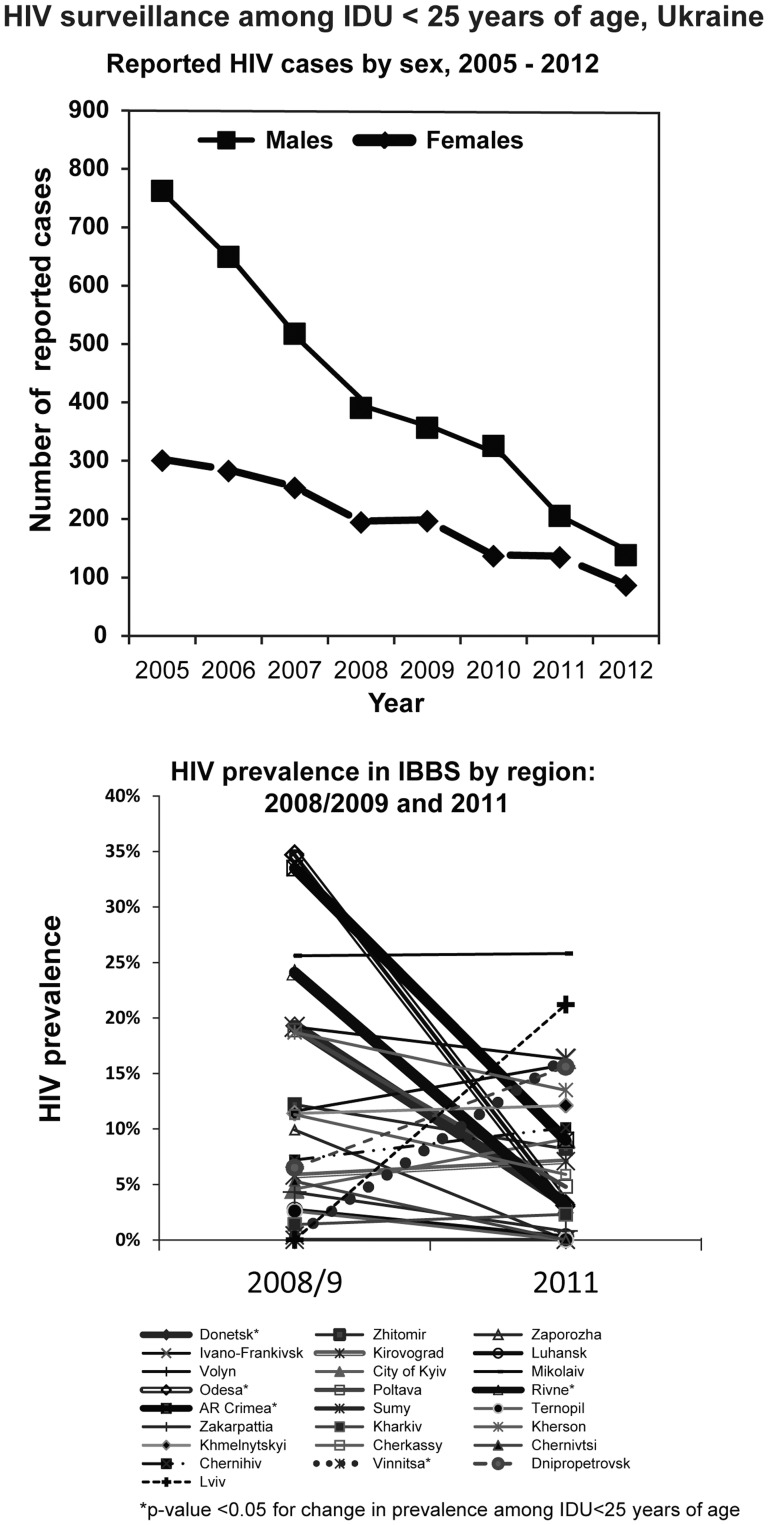
HIV surveillance among IDU<25 years of age, Ukraine. a. Reported HIV cases by sex, 2005–2012. b. HIV prevalence in IBBS by region: 2008/2009 and 2011.

From 2005–2012, 54,053 (44.9%) newly reported cases of HIV were attributed to injection drug use (IDU), and, of these, 80% were male. Among individuals <25 years of age, 4,884 HIV cases were attributed to IDU. Nationwide, the number of annually reported cases among PWIDs<25 years of age fell by 78% from 2005 to 2012 (**Figure 4a**).

Overall HIV prevalence among PWID as measured in IBBS remained unchanged at 21.6% between 2009 and 2011. Among PWID<25 years old, HIV prevalence declined from 9.9% to 7.2% overall (P = 0.01), with declines in almost all regions with higher incidence among PWID (**Figure 4b**)[12]–[14].

### Trends in reported HIV cases attributed to heterosexual transmission

From 2005–2012, 63,177 cases of HIV were attributed to heterosexual intercourse, and of these 62% were women and 38% men.

#### Women

The number of annually reported cases attributed to sexual transmission in women ≥25 years old more than doubled from 1,814 cases in 2005 to 5,057 cases in 2012. Among younger women, HIV cases attributed to heterosexual transmission peaked in 2008 with 297 cases (1.9 cases per 10,000 population) among 15–19 year olds and 1,226 cases (6.4 cases per 10,000) among 20–24 year olds; in 2012, 177 cases (1.4 cases per 10,000) were reported among 15–19 year olds and 910 cases (5.5 cases per 10,000) among 20–24 year olds, representing 24.4% (p<.01) and 14.7% (p<0.001) declines in the rates of reported cases in the respective age groups. The largest declines in heterosexual cases in women <25 years old were in the nine regions with the highest incidence of reported HIV cases among PWID, with declines of 40% in Donetsk and Mykolaiv oblasts and 65% in Kyiv City.

#### Men

From 2005 to 2012, the annual number of adult and adolescent cases attributed to sexual transmission increased by <1% among men <25 years old but increased by 202% among men ≥25 years old. A 2008–2009 study surveyed newly registered male HIV cases attributed to heterosexual transmission and found that 8.5% reported a history of personal IDU and 17.3% reported PWID sexual partners; reviews of case records found that 27% had HCV antibodies. The study did not report on the overlap between antibody status and reported risk factors [15].

### Trends among pregnant women

**Figure 5:**
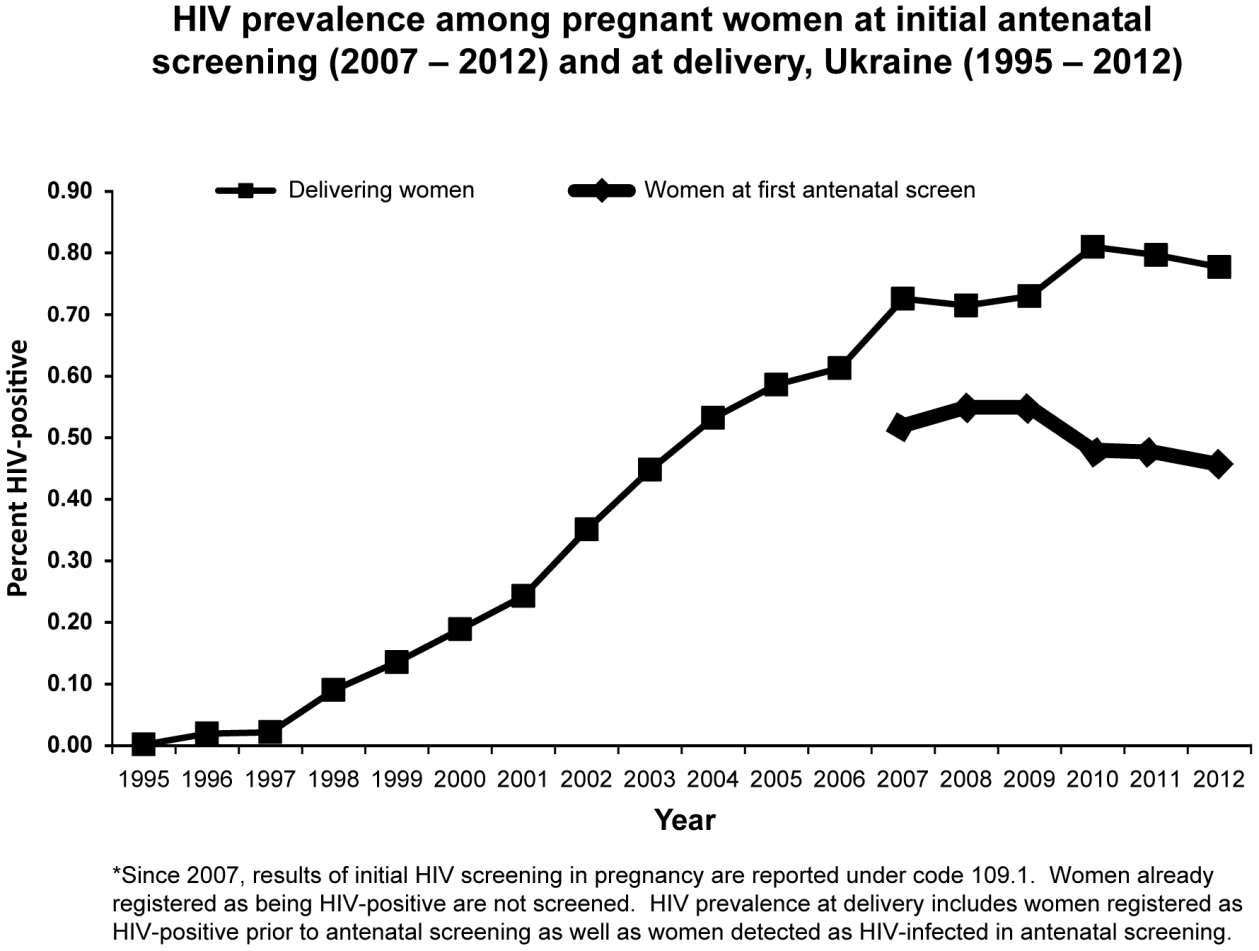
HIV prevalence among pregnant women at initial antenatal screening and at delivery, Ukraine 1995–2012.

HIV prevalence among all women who delivered rose progressively from 0.59% in 2005 to 0.81% in 2010 (6.2% per year, 95% CI: 5.4% to 7.1 %) and then stabilized, being 0.78% in 2012 (−1.7% per year, 95% CI: −3.6% to 0.1%, from 2010 to 2012); the trends in these two periods were significantly different (p<0.01) (**Figure 5**). The overall HIV prevalence found in initial ANC screening of pregnant women who were not previously registered as HIV-infected rose from 0.52% in 2007 to 0.55% in 2009 (1.6% per year, 95% CI: −0.7% to 3.9%), and then decreased to 0.45% in 2012 (−6.8% per year, 95% CI: −8.3% to −5.4%); the trends in these two periods were significantly different (p<0.01). HIV prevalence in initial ANC screening correlated with incidence among PWID; median prevalence was 0.73% in the PWID cumulative case-report top tercile of regions, 0.25% in the middle tercile, and 0.12% in the low tercile. A survey among 839 HIV-infected women detected in ANC from 2009 to 2011 found that 6.6% reported personal histories of IDU while 24.8% reported a PWID partner [15].

### HIV prevalence in men entering military service

Universal HIV screening of male conscripts began in 2008. As of 2012, 340,405 men had been screened, and 284 (0.08%) were found to be HIV-infected. There has been a gradual decline in prevalence among male conscripts over this period, from 0.11% in 2008 to 0.07% in 2012, a decline of 13.7% per year (95% CI: 4.8%–21.7%). In a 2007 survey of conscripts, 1.5% reported having ever injected drugs and 0.4% had injected in the last month [16].

### HIV prevalence in FSW and clients of FSW

In the 2011 IBBS, an estimated 9.0% of FSW were infected with HIV, compared to 12.9% in 2009 [17]–[18]. Among FSW 14–24 years old, the prevalence declined from 8.3% to 3.6%; both declines were statistically significant (p<0.01) [18]. HIV prevalence among FSW was generally higher in the highest PWID tercile oblasts (Table 1) and infection status among FSW was associated with a personal history of IDU. In the 2011 survey, overall HIV prevalence among FSW was 41.1% among those who reported having injected drugs in the last 12 months, 31.3% among those who reported injecting but not within 12 months, and 6.0% among those who denied ever using drugs (p<0.01 for all comparisons [18]). Among FSW who denied drug use but who were HCV-antibody positive, HIV prevalence was 30.3%. Of 1,117 FSW who either admitted to drug use or were HCV-seropositive, 346 (31.1%) were seropositive compared with 156 (4.1%) of 3,846 HCV-antibody negative FSW who denied drug use (odds ratio 10.7; 95% CI 8.7–13.1).

**Table 1:** Reported HIV cases by region and attributed mode of transmission, Ukraine, 2005–2012 and mean prevalence in integrated biobehavioral surveys (IBBS) among key populations.

Region	Population (2010)	Reported IDU HIV cases	Mean annual IDU incidence*	Reported hetero-sexual HIV cases	Mean annual hetero-sexual incidence*	IDU 2011 IBBS [ref. 10]		FSW 2011 IBBS [ref. 13]		MSM 2011 IBBS [ref. 16]	
						HIV Preval-ence	95% CI	HIV Preval-ence	95% CI	HIV Preval-ence	95% CI
Dnipropetrovsk	3,355,500	10,207	**38.0**	9,186	34.2	33.4%	28.1%–39.2%	9.6%	6.2%–12.9%	5.4%	3.0%–7.8%
Mykolaiv	1,189,500	3,013	**31.7**	3,955	41.6	40.2%	25.1%–45.9%	7.1%	4.2%–10.0%	2.0%	0.6%–3.4%
Donetsk	4,466,700	10,302	**28.8**	14,112	39.5	20.9%	16.6%–25.5%	42.7%	33.9%–53.2%	20.2%	16.2%–24.1%
City of Sebastopol	380,500	787	**25.9**	665	21.8	–	–	–	–	7.3%	3.1%–11.5%
Kherson	1,093,400	1,753	**20.0**	1,919	21.9	28.4%	23.1%–34.2%	9.3%	5.3%–13.3%	5.4%	2.6%–8.2%
Odesa	2,391,000	3,852	**20.1**	6,853	35.8	32.0%	27.9%–36.4%	13.5%	9.7%–17.4%	16.2%	12.5%–19.8%
City of Kyiv	2,785,100	4,307	**19.3**	2,923	13.1	25.8%	17.4%–33.1%	24.2%	18.1%–30.6%	6.9%	4.4%–9.4%
AR Crimea	1,965,300	2,668	**17.0**	3,558	22.6	22.6%	18.8%–26.4%	3.6%	1.5%–5.6%	2.6%	0.4%–4.8%
Kyiv	1,721,800	1,718	**12.5**	2,019	14.7	–	–	–	–	–	–
Chernihiv	1,109,700	1,061	**12.0**	1,293	14.6	33.1%	27.2%–38.9%	1.0%	0.0% 4.6 %	1.4%	0.0%–3.3%
Cherkassy	1,295,200	1,156	**11.2**	1,349	13.0	26.2%	21.4%–31.0%	14.4%	6.5%–24.0%	3.0%	0.9%–5.2%
Luhansk	2,311,600	1,873	**10.1**	2,305	12.5	2.4%	1.1%–3.9%	0.0	–	5.9%	2.6%–9.2%
Poltava	1,499,600	1,234	**10.3**	1,262	10.5	22.8%	17.1%–28.4%	26.5%	20.4%–32.6%	0.1%	0.0%–0.6%
Zhitomir	1,285,800	952	**9.2**	1,174	11.4	19.0%	14.9%–23.1.%	5.3%	1.7%–8.9%	11.9%	6.7%–17.1%
Zaporozhe	1,811,700	1,331	**9.2**	1,779	12.3	5.8%	2.0%–10.4%	4.8%	1.9%–9.1%	5.1%	2.0%–8.2%
Kharkiv	2,769,100	1,812	**8.2**	1,322	6.0	8.4%	5.3%–12.0%	0.0	–	4.4%	2.1%–6.7%
Khmelnytskyi	1,334,000	788	**7.4**	877	8.2	33.7%	28.7%–40.4%	18.7%	12.4%–24.9%	7.8%	3.5%–12.2%
Vinnitsa	1,650,600	926	**7.0**	1,195	9.0	13.0%	9.2%–16.9%	1.5%	0.0%–3.5%	6.1%	2.2%–10.0%
Lviv	2,549,600	1,371	**6.7**	895	4.4	27.6%	21.7%–34.1%	5.7%	2.5%–8.9 %	6.8%	3.6%–9.9%
Rivne	1,151,600	607	**6.6**	559	6.1	9.2%	6.1%–12.6%	4.8%	1.4%–8.2%	2.0%	0.0%–4.2%
Kirovograd	1,017,800	495	**6.1**	1,027	12.6	9.0%	4.9%–13.2%	13.7%	8.2%–19.2 %	3.9%	0.8%–7.1%
Volyn	1,036,700	445	**5.4**	867	10.5	18.0%	13.7%–23.5%	5.2%	1.6%–8.7 %	1.6%	0.0%–3.7%
Ternopil	1,088,900	460	**5.3**	338	3.9	17.2%	8.7%–24.9%	2.0%	0.0%–4.2%	1.3%	0.5%–3.2%
Sumy	1,172,300	473	**5.0**	608	6.5	4.2%	2.1%–6.7%	0.9%	0.9%–2.7%	5.4%	2.3%–8.6%
Ivano-Frankivsk	1,380,700	316	**2.9**	506	5.1	16.9%	11.3%–22.4%	9.8%	5.0%–14.5%	6.3%	2.3%–10.2%
Chernivtsi	904,400	131	**1.8**	380	5.3	3.7%	1.3%–6.6%	2.0	0.0%–4.3%	2.5%	0.0%–5.0%
Zakarpattia	1,244,800	15	**0.2**	251	2.5	1.3%	0.0%–2.6%	0.0	–	5.2%	1.6%–8.8%
Total**	45,962,900	53,051	**14.7**	63,177	17.2	21.6%		9.0%		6.4%	

*Cases per 100,000 total population.

**Confidence intervals were not reported for overall national prevalence estimates in the 2011 IBBS.

AR Crimea, Autonomous Republic of Crimea.

A survey of clients of FSW in 2009 showed an overall prevalence of 7.4%. The prevalence was 23% among clients admitting previous IDU and 3% among those clients denying IDU [19].

### HIV in MSM

Only 645 cases from 2005–2012 were attributed to male-to-male sexual transmission. The 2009 and 2011 IBBS found an overall HIV prevalence of 8.6% and 6.4%, respectively among all MSM nationwide and 7.4% and 4.2% respectively among MSM<25 years of age [20]–[21].

## Discussion

Although UNAIDS had previously included Ukraine as part of the growing Eastern Europe and Central Asia HIV epidemic [22], the 2013 UNAIDS Global Report indicates there are an estimated 230,000 PLHIV in 2013 and that modeled incidence has declined 68% from 2001 to 2012 in Ukraine [5]. The marked decline in prevalence from the 2007 estimate [2] reflects improved data for modeling as well as the impact of decreased incidence and substantial mortality. Our study also indicates that the epidemic among PWID in Ukraine is declining, that the female heterosexual epidemic linked to the PWID epidemic [23] may have peaked, and that the available evidence suggests a declining incidence in recent years. The case reporting data show that overall cases have begun to decline in 2012 while older, more clinically advanced patients are responsible for all of the increase in cases seen from 2005 to 2011. The increasing age and immunodeficiency at presentation of these cases almost certainly reflect the detection of patients who were infected long ago and who presented due to clinical need. The progressive decline in new HIV cases with asymptomatic illness since 2009 suggests a decline in the number of undiagnosed individuals in earlier stages of illness.

The declines in both reported cases and HIV prevalence among young PWID in IBBS also support a decrease in transmission among PWID. The plateauing and subsequent modest decline in HIV cases among all PWID since 2005 and the recent decline in overall cases suggests that a majority of the individuals infected in the early large wave of epidemic spread from 1995–1998 have either been diagnosed (and registered) or died. Mortality analyses show a disproportionate mortality burden among PWID, with late presentation and inadequate access to ART as contributing factors [24].

The overall number of HIV-infected PWID remains high, and this predominantly male group continues to be a major source for new heterosexual infections, especially in female partners. This is supported by the higher prevalence among women in regions with higher HIV prevalence among PWID and by the large proportion of HIV-infected women newly detected in ANC screening who, despite considerable stigma, report IDU as a risk factor in their partners.

The decline in reported HIV cases among women younger than 25 years may suggest that heterosexual transmission has begun to decline. Screening of pregnant women has also shown a decline in the prevalence of newly detected HIV infections. The pregnancy screening indicator in Ukraine is likely to be affected quickly by changes in incidence in the underlying population of women, since previously registered HIV cases are excluded, screening in pregnancy is essentially universal among women preventing for ANC care, and because the low coverage of hormonal contraception [25] should result in a high rate of pregnancy among women acquiring HIV heterosexually. We postulate that the decline in cases among young women is causally related to declining transmission from infected PWID to their partners.

The survey data clearly show the strong relationship between IDU and increased risk of HIV in both FSW (by history and by HCV antibody status) and their clients (by history) in Ukraine. The true contribution of IDU to HIV infection in these groups is likely underestimated due to self-underreporting of IDU. Even with the use of HCV status to indicate a history of IDU, not all PWID will be identified, since not all are HCV seropositive. In the 2011 IBBS, only 61% of HIV-infected female FSW who admitted IDU were HCV seropositive, so the HIV seroprevalence among FSW who have truly never injected is almost certainly less than the 4% observed among those who denied injecting and who were HCV seronegative. These data from FSW and conscripts that indicate much of the observed HIV prevalence in both groups could be accounted for by IDU, suggest limited HIV transmission that is not linked to IDU in these groups.

In contrast, reported cases among men attributed to heterosexual transmission have risen sharply, primarily among those older than 30 years. We believe that misclassification contributes significantly due to the stigma associated with IDU and MSM behavior. The history and hepatitis C antibody status of the male ‘heterosexual’ cases reviewed demonstrated that >25% had evidence of personal or sex partner IDU behavior. Other historical evidence of misclassification of male cases due to IDU includes data from the case-reporting system from 1995–1996, showing that among the 5,004 adult male cases reported, 752 cases were attributed to ‘heterosexual’ transmission and 276 to ‘other’ causes. During this period of initial intense IDU transmission, only infrequent instances of heterosexual transmission to males would have occurred. Presumably, this misclassification by the registering physicians reflected less obvious IDU behavior among these individuals; individuals with less intense IDU behavior would also be likely to have decreased contact with the HIV screening sites that access males, which are primarily in drug treatment, prison, and clinical facilities. A greater proportion of these ‘misclassifiable’ IDU cases might therefore be detected only later with onset of clinical symptoms; such cases may be contributing to the increase in recent years in newly reported older ‘heterosexual’ cases with advanced immunodeficiency. Additionally, significant misclassification of MSM in case reporting is supported by the contrast between low numbers of reported cases among MSM and the MSM IBBS prevalence data [26].

There are several limitations in interpreting the data analyzed here. All HIV case reporting systems are dependent on case presentation, detection, registration, and reporting; collection of risk factor data in these systems varies greatly. In Ukraine, the case reporting is facilitated by a centralized system of HIV care and reporting functioning since 1987 with only limited changes. The dominance of government health care and the existence of severe administrative penalties for nonreporting also reinforce complete reporting; epidemiologists from the Ukrainian Center for Socially Dangerous Disease Control who monitor AIDS Centers' surveillance functions consider reporting of cases to be highly complete. However, risk behavior data are limited to presumed mode of transmission. Even these data are either underreported (PWID, MSM) or not collected (FSW) and analysis has not routinely triangulated data from other studies where more behavioral data were available. HIV screening and confirmation is limited to state-certified laboratories and these data are also reported in a standardized fashion. Assessments by UNAIDS of the Ukrainian case reporting surveillance system [27] and the similar Russian system [28] support these strengths and limitations. Another limitation is that ANC screening, although extremely high among pregnant women presenting for antenatal care has been incomplete (50%–60%) for women presenting for therapeutic abortion during this period; these women represented 21.4% of all pregnancies in 2012. However, the progressive and highly significant decline in both relative and absolute number of HIV-positive women found in screening from 2008–2012 suggests that random fluctuation in testing among the unscreened minority of women presenting for abortion is unlikely to be responsible. In addition to these general limitations, the population screening and case reporting system in Ukraine is likely to detect HIV cases earlier among women than men, as a significant proportion of women infected heterosexually will be detected soon after infection given the low prevalence of hormonal contraception and near-universal ANC screening of pregnant women presenting for antenatal care. The lack of comparable screening access to men means that a higher proportion of infected men will be detected later in their disease. Another limitation of routine data is the inability to differentiate whether women with heterosexually acquired HIV were infected by PWID or by non-PWID partners. In addition, comparisons of numbers of case reports from younger age cohorts are becoming more complicated by the aging into adulthood of birth cohorts born after 1991, when an unprecedented decline in births followed the breakup of the Soviet Union [29]. However, the observed declines in cases among young females exceed the declines in population, resulting in significantly lower rates of reported cases. Also, the declines in HIV prevalence measured in ANC screening and in IBBS among key populations would not be affected by declining population size and provide independent support for our finding of a decline in HIV among younger age groups. While IBBS surveillance data from the earlier rounds are limited by lack of consistent coverage of all regions and by variation in methodology for FSW studies, the prevalence declines are also found in the 2009 and 2011 rounds, which used comparable methodology and achieved national coverage for PWID and FSW.

Modifications of HIV surveillance in Ukraine are needed to more effectively track the evolving HIV epidemic. Periodic studies to elucidate the risk status of partners of newly reported female cases, especially among younger women, would help in tracking further evolution of the epidemic, as would studies to better identify risk behavior histories for reported male heterosexual cases. Additional studies to explore sexual mixing patterns among PWID and non-drug-injecting heterosexual ‘‘bridge populations’ would also be useful [23]. Finally, new assays for recent HIV infection, such as the limiting antigen assay [30], should be incorporated into surveillance activities to characterize risk factors for recent infection and more accurately track incidence in risk groups.

From the programmatic viewpoint, enhancing prevention among KP remains the highest priority. Given the continuing critical role of IDU, further scale up of coverage with needle and syringe exchange programs, opioid substitution therapy [31], and ART for HIV-infected PWID at earlier stages of infection should be priorities. Given high HIV prevalence among FSW, earlier identification and treatment of HIV-infected FSW may offer an opportunity to further limit heterosexual transmission. The persistent risk behavior and limited coverage of prevention programs among MSM [21] raise concerns about expansion of this epidemic, and increased prevention efforts are needed. Expanded and enhanced prevention efforts, including identification of discordant couples and increased ART, could help limit transmission between infected PWID (and former PWID) and their partners and help address a major source of infection for young women [32].

Increased efforts in Ukraine to enhance prevention and treatment among KP are still needed, combined with strong advocacy to enhance government support of prevention programs and decrease dependency on external funding. Thesemeasures could limit further growth in the Ukrainian epidemic but will be extremely challenging in the short term, given recent decreases in Global Fund resources for Ukraine and the severe strain on government resources due to the ongoing armed conflict in Eastern Ukraine. However, Ukraine's success in expanding prevention coverage among KP and the evidence of significant improvements in the epidemic to date stand in contrast to the situation in the Russian Federation where ‘Western’-style harm reduction efforts are not prioritized [33], reported cases are accelerating [34], and the WHO/UNAIDS estimate exceeds 900,000 PLHIV in 2013 [5]. These trends support the effectiveness of prevention programs among KP in Eastern Europe and suggest that Ukraine may no longer have the most severe HIV epidemic in Europe.
